# Narrative Review: Sugar and Rice and the Diabetes Epidemic in India—A Historical Context

**DOI:** 10.3390/nu18121973

**Published:** 2026-06-18

**Authors:** Shaminie J. Athinarayanan, Desmond D. Mascarenhas, Balaji Rajagopalan, John W. Fox, Miguel A. Lanaspa, Richard J. Johnson

**Affiliations:** 1 Research Department, Virta Health, Denver, CO 80216, USA; 2 Department of Research, Mayflower Organization for Research & Education, Sunnyvale, CA 94085, USA; 3 Department of Civil, Environmental and Architectural Engineering, and CIRES, University of Colorado, Boulder, CO 80309, USA; balajir@colorado.edu; 4 Department of International Studies, American University of Sharjah, Sharjah P.O. Box 26666, United Arab Emirates; 5 Department of Medicine, University of Colorado Anschutz Medical Campus, Aurora, CO 80045, USA

**Keywords:** type 2 diabetes, sugar, fructose, high glycemic carbohydrates, kwashiorkor, protein malnutrition, obesity, insulin resistance

## Abstract

South Asians appear to be particularly susceptible to diabetes. India hosts 18 percent of the world’s population but more than 25 percent of the world’s diabetics, and individuals of South Asian descent carry this presumed increased risk for diabetes when they emigrate to other parts of the world. One conundrum is that the epidemic of diabetes began around Calcutta (modern day Kolkata) in east India well before it appeared in the United States and Europe, and this emergence occurred despite the frequent occurrence of famines and starvation in India. Here we review the history of diabetes in India and the possible significance of high carbohydrate in low-protein diet contexts. We suggest that the circumstantial relationship between diet and a spectrum that includes diabetes associated with obesity at one end, and impaired glucose tolerance and protein malnutrition (kwashiorkor) at the other, could be significant. If the cause of type 2 diabetes in South Asians is primarily nutritional, and, as suggested by others, aggravated by starvation and famine that increased the risk for low birth weight as an additional risk factor for diabetes, these insights may together help explain an enhanced susceptibility of South Asians to diabetes.

## 1. Introduction

“What gout is to the nobility of England, diabetes is to the aristocracy of India” CL Bose, Medical Examiner, State of Bengal 1907 [1].

While there are many genetic and environmental risk factors that increase the susceptibility of individuals to develop type 2 diabetes, one of the potential contributors is a diet high in sugar (sucrose) and high-glycemic-enriched carbohydrates [2,3]. Epidemiological studies have linked the introduction of sugar into indigenous populations with the appearance and rise in diabetes [4,5,6,7]. A classic and carefully performed epidemiological study by New York City Health Commissioner Haven Emerson reported that a remarkable rise in type 2 diabetes began at the turn of the twentieth century in New York City, where it was strongly linked with increased sugar intake, and that diabetes was less frequent in other countries such as Australia where sugar intake was much lower [8]. The administration of sugar was also shown to induce type 2 diabetes in laboratory animals, and this could occur even with dietary caloric restriction [9].

Further studies showed that it was the fructose component of sucrose that was particularly capable of inducing insulin resistance, and this was associated with a unique metabolism linked with the stimulation of glycolysis, de novo lipogenesis, oxidative stress to the mitochondria, and uric acid generation [10,11,12]. While fructose can induce these metabolic effects in the absence of increased energy intake [13], fructose ingestion in animals also blocks satiety by inducing leptin resistance; hence, many of the effects of sugar intake on metabolic disease are enhanced from increased food intake and weight gain [14,15]. High-glycemic carbohydrates can also induce insulin resistance and chronic hyperinsulinemia, but while the direct stimulation of insulin may be partially involved, the major mechanism appears to be from the intrahepatic conversion of glucose to fructose via the polyol pathway [16,17].

Clinical studies in humans have confirmed the ability of fructose (and, to a lesser extent, glucose) to induce insulin resistance as well as other features of metabolic syndrome [18,19], and also show that sugar (and/or fructose) restriction can improve metabolic features [20,21]. Ketogenic diets in the absence of caloric restriction have also been reported to improve insulin resistance and prediabetes in subjects with metabolic syndrome [22]. Confirming these findings, epidemiological studies have shown that the recent reduction in the intake of sugary beverages is paralleled by a decreasing incidence of type 2 diabetes (an observation made even before GLP1 agonists were introduced) [23].

While the biological evidence for sugar and high-glycemic carbohydrates as contributing risk factors for diabetes is strong, evidence from other disciplines is useful. One approach is to evaluate the relationship between a change in diet within a country and the rise in diabetes. In this regard, India is of special interest as it is here where sugarcane was first domesticated, and where some of the earliest reports of diabetes were noted [24]. Today, India is the “diabetes capital of the world” [25] with over 200 million people with diabetes, accounting for more than one-quarter of all subjects with diabetes globally [26]. Yet there was a time when diabetes was exceptionally rare [27]. Recent large-scale epidemiologic analyses further demonstrate substantial geographic heterogeneity in diabetes prevalence across India, with clustering in southern and coastal regions and associations with behavioral and socioeconomic factors such as obesity, alcohol use, and tobacco exposure [28].

Compared with many other populations, South Asians—particularly Indians—develop type 2 diabetes at younger ages and lower body mass indices and frequently exhibit increased visceral adiposity despite relatively low overall adiposity [29,30,31,32]. Emerging evidence suggests that this susceptibility reflects a combination of genetic predisposition, reduced β-cell reserves, unfavorable fat distribution, developmental programming, and the broader “South Asian phenotype” [29,31,32]. Recent genetic studies also indicate that South Asians carry a greater burden of genetic variants associated with insulin deficiency and adverse fat distribution, which may contribute to earlier diabetes onset and more rapid disease progression [33]. Nevertheless, genetic factors alone cannot explain the dramatic increase in diabetes prevalence observed during the last century. Rather, inherited susceptibility likely interacts with environmental exposures, including dietary transitions, urbanization, reduced physical activity, and socioeconomic change, to amplify diabetes risk in South Asian populations [29,31,32].

Here we present a historical and archeological multidisciplinary perspective on the relationship between sugar and rice intake and the rise in diabetes in India. We also discuss the role of low protein intake, the impact of famines, the role of low birthweight and fetal malnutrition, and the appearance of “type 5” diabetes. The historical account is surprisingly consistent with the hypothesis that a parallel rise in sugar and white rice consumption created an unusual low-animal-protein nutritional context within which the high prevalence of diabetes in South Asia is better understood. The circumstantial evidence from a different discipline cited here is thus consistent with the hypothesis that excess fructose within a unique nutritional context leads to a higher incidence of diabetes in South Asians.

### Sugarcane Before and During the British Occupation of India

While sugarcane was likely domesticated in New Guinea, India was the first region where it was extensively processed into sugar. Sugarcane could be chewed directly, pressed to obtain juice, or boiled to produce jaggery, an unrefined sugar that was widely consumed throughout South Asia. Archeological evidence suggests sugarcane use during the Harappan civilization (2600–1900 BCE), where phytoliths of sugarcane and date palms have been identified at multiple sites [34]. In addition, dental caries rates among Harappan populations was substantially higher than among their Neolithic predecessors, suggesting greater exposure to dietary sugars [35].

The earliest historical reference to sugarcane is in the Rigveda of the Vedic period (ca.1000 BCE), when it was fed to a horse slated for ritual sacrifice [36]. Sugarcane was used both as an Ayurvedic medicine and food. It was desired for its sweetness, as one passage states, “Like sweet juice from the sugarcane (ikṣu), the pressed Soma flows” (Rigveda 9.67.31) (Soma was a sacred, psychoactive drink of unknown composition). A multitude of legends and myths exist about its sweetness [36]. For example, Kamadev, the Indian Cupid, was said to have a bow of sugarcane with a string of bees and honey, and with the arrows tipped with the flowers of Vasanta. “He bends the luscious bow and twists the string, With bees so sweet, but ah, how keen the sting! He with fine flowers tips the ruthless darts, Which through five senses pierce enraptured hearts” [36].

Ancient Ayurvedic physicians were among the first to recognize a possible relationship between sweet foods and metabolic disease. Sushruta (circa 180 CE) described obesity and diabetes (madhumeha) and linked these conditions to the consumption of sweet foods and rice combined with sedentary lifestyles [24,37,38,39]. He even described a form of diabetes termed “ikshumeha,” referring to urine resembling sugarcane juice. Similarly, Charaka noted the ability of sugarcane to promote nutrition and corpulence [36]. These observations represent some of the earliest recorded associations between diet and diabetes.

During the Roman Warm Period (250 BCE~400 CE), also the period associated with Sushruta (~180 CE), the Indian monsoon was stronger than today, making already wet regions of the Indo-Gangetic Plains in central and western India even wetter [39]. This humid phase supported agricultural prosperity and societal growth. Favorable monsoon conditions likely increased the cultivation of water-intensive crops such as sugarcane and rice, particularly across the Indo-Gangetic Plains, long known as India’s “sugar bowl.” Enhanced wetness also characterized the Medieval Warm Period (905–1250 CE), along with shorter wet phases in the late 1600s and early 1800s during British rule, which likely supported expanded sugarcane cultivation and the establishment of sugar mills. Overall, multi-decadal to centennial monsoon variability over the past millennium [39] played an important role in sustaining large-scale sugarcane and rice production and their integration into regional culture.

Sugarcane was little known outside India until Alexander the Great reached the shore of the Indus River. His admiral, Nearchus, called it “honey without bees”. During this period, jaggery was produced by farmers, primarily in the Ganges and Indus River valleys [40], where it was used in specific desserts as opposed to regular foods [36]. Jaggery was also made from palm sap in Tamil Nadu and Ceylon (Sri Lanka). While the quality of the jaggery from palms was inferior to jaggery from sugarcane, it was more widely available [40].

Jaggery was initially exported by boat to Persia and Mesopotamia, and traded overland to Persia and China. By 600 CE, Arabs had expanded the range, cultivating sugarcane to Egypt and Cyprus, and eventually producing raw sugar rather than jaggery. Within a little more than a century, sugarcane was grown along the entire northern coast of Africa (Magreb) to Iberia (al-Andalus). But while sugarcane became a profitable trade elsewhere, production remained relatively low in India.

In the beginning of the modern era, Europeans, and especially the English, sought sugar from the West Indies. The Dutch and British soon realized the potential for sugar production in Asia and founded the competing Dutch East Indies Company in 1602 and the British East India Company in 1600. Both soon developed sugar plantations and sugar mills to refine sugar for export to Europe [36], with the Dutch building sugar mills in Taiwan and Java, while the British built mills in India, with the first sugar mill being in the Coromandel Coast of southeast India in 1610, and the second in Surat (in the state of Gujarat) in 1612. However, the early British mills were not very successful as some produced sugar from palm sap (a lower-quality sugar) and because the growing of sugarcane carried a high requirement for water that limited the regions where sugarcane could be produced [40]. England also stymied production by imposing a tax on sugar from the East Indies, which favored sugar production to the West Indies [36,40]. For these reasons, crystalline sugar production from cane in India remained limited.

Even though the production of sugarcane was low, a famous Scottish physician by the name of Thomas Christie published a report in 1811 of a surprisingly high frequency of diabetes among natives in Ceylon [27]. Christie described 10 cases of diabetes and noted there was a strong linkage with a diet high in palm sugar, fruit, and rice while simultaneously low in protein. He specifically pointed out that some individuals were obese while others had low body weight and were debilitatingly weak [27]. In attempting explanation through comparisons, he noted there was an almost complete absence of diabetes in Calcutta (the capital), where there was an absence of sugar in the diet. He also noted that diabetes in Ceylon could be improved by avoiding sugar and eating a diet high in animal protein [27].

**Figure 1:**
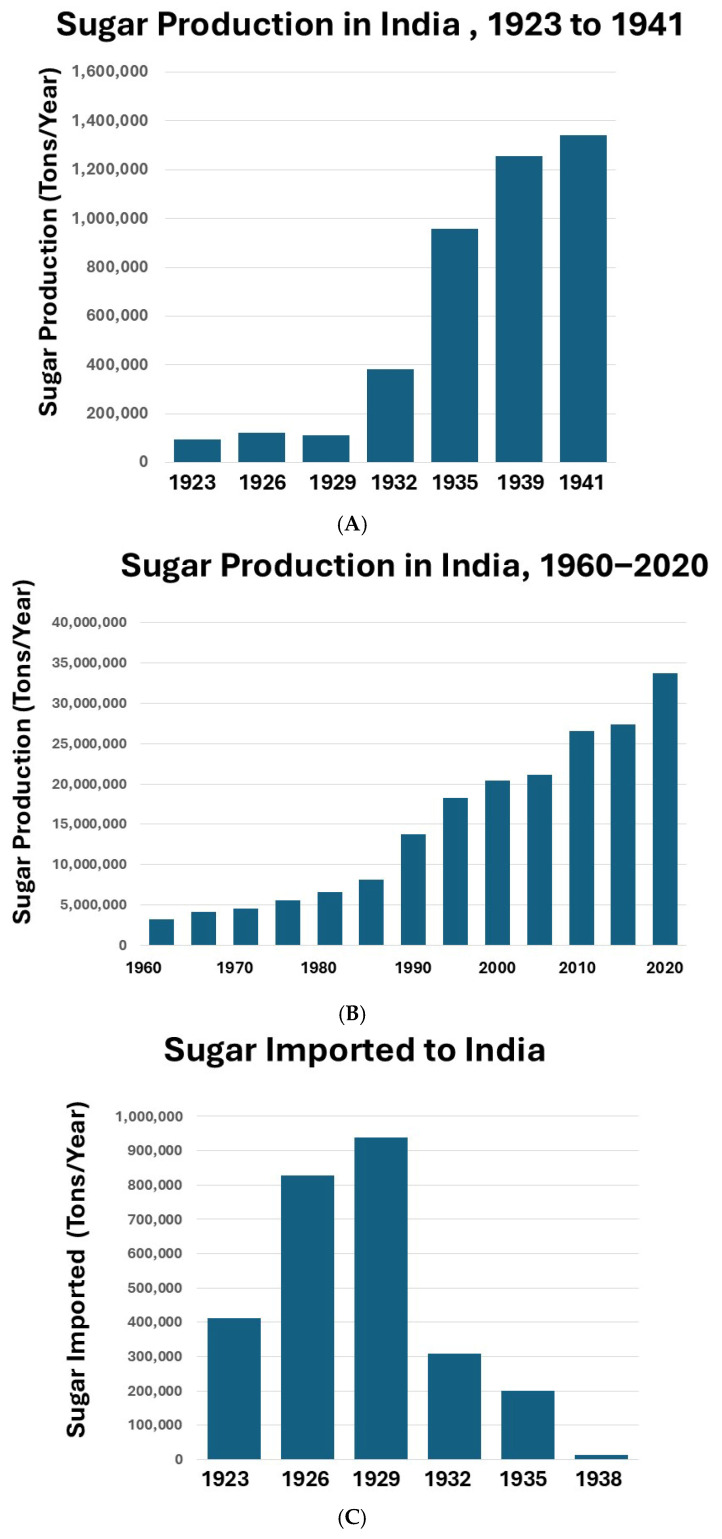
Sugar trade in India. (**A**) The dramatic rise in sugar production in India during the 1930s. (**B**) Sugar production increased to over 30 million tons by 2020. (**C**) The importation of sugar rapidly declined as production increased (**B**). Data for (**A**,**C**) from [31]. Data for (**B**) is from the United States Department of Agriculture at https://www.indexmundi.com/Agriculture/?country=in&commodity=centrifugal-sugar&graph=cane-sugar-production (accessed on 6 June 2025).

As British colonization increased, India could not keep up with the local demand for sugar, and India began to import it. This led to a more vigorous attempt to increase the number of sugar mills in India. By the late 1700s, sugar mills were built in Hughli, West Bengal, near Calcutta. Production further increased when the duties for East Indian sugar were finally abolished in 1836. By the late nineteenth century, sugar was more readily available to the wealthier castes, such as the Brahmins [40]. Sugar went from being a rare luxury to a basic commodity, especially in Calcutta and the surrounding Bengal State. The upper-strata Indian classes variously emulated the British overlords in habit and taste. Sugar mills expanded to Madras (modern day Chennai). Within decades, India exported considerably more sugar than it imported. By the 1930s, the importing of sugar was ending while production was skyrocketing. By 2020, over 30 million tons of sugar were being produced per year (Figure 1) [36].

Just as the emergence of obesity and diabetes in Europe was first reported in England and Holland, where the sugar was being imported [41,42], diabetes was also first noticed in the regions of India where sugarcane was being grown. As early as 1867, diabetes was reported in Bengal State, where it was linked with an intake of fat and “sweets” [43]. By 1880, diabetes was becoming increasingly observed in Calcutta [44] where it occurred in the educated and rich (especially the Brahmins) and was associated with diets high in rice and sugar and low in fish, milk and dal (such as lentils). Mitra, a physician who himself had diabetes, noted over 350 cases in India in 1896 [45]. While the prevalence of diabetes in Europe and America was only present in two to three cases per 100,000 people [46], Bose stated, based on his personal experience, that nearly 10 percent of well-to-do Bengali suffered from the disease [47]. Of interest, diabetes was observed primarily in Bengal, while Punjab still had very little diabetes [48]. Of note is that diabetes was rare or absent in Calcutta only 80 years before [27].

A conference in 1907 in Exeter discussed this epidemic of “tropical diabetes”. The primary group developing diabetes were wealthy or educated Hindus whose diet was mostly sugar and starch, especially rice, and with very little animal protein because they were often strictly vegetarian [1,47,48,49]. A similar rise in diabetes was occurring in Ceylon where the diet amounted to >3000 calories/day of carbohydrate and only 120 to 240 calories of protein [49]. Egypt also reported increasing cases of diabetes. One of the more striking issues was that diabetes was often indolent, lasting 20 years or more, and was associated with the development of cirrhosis [47], kidney disease and angina pectoris [47]. The description of cirrhosis was likely one of the earliest reports of metabolic dysfunction-associated steatotic liver disease (MASLD).

In addition to sugar facilities, the British built rice mills in Bengal and Madras where white, polished rice was increasingly consumed despite being devoid of minerals, vitamins (i.e., thiamine) and protein. By 1927, the prevalence of diabetes reached 6.5 percent of all admissions in Madras, 4.2 percent in Bengal, and less than 2.4 percent in the other provinces [50].

Treatment typically involved reducing sweet and starchy carbohydrates, and encouraging milk (as a source of protein), butter and vegetable intake [51,52]. Physicians considered this treatment effective since it reduced the excess loss of glucose in urine. It was argued that Muslims ate greater amounts of animal protein that explained why they had a lower frequency of diabetes [48].

Since the 1920s, diabetes rates have increased in India, as well as in Islamic countries such as Pakistan (30%) and Kuwait (25%) [53]. Currently, the prevalence of diabetes is higher among the poorer population in urban areas, though higher in the wealthier segments in rural areas [54]. However, large nationally representative analyses indicate that the overall burden of diabetes in India remains disproportionately concentrated among higher socioeconomic groups, with 70–90% of cases occurring in higher wealth, education, and caste strata [55]. One potential explanation is that sugar and soft drinks are sufficiently inexpensive and available to the poor in the cities, whereas soft drinks are beyond the means of the impoverished in rural areas for habitual consumption. Another contributing factor may be dietary patterns among higher socioeconomic groups, which often include a higher intake of refined carbohydrates such as polished rice and added sugars, and, in some cases, lower intake of animal-source protein due to vegetarian dietary practices, potentially exacerbating carbohydrate-driven metabolic risk. Likewise, the continued high prevalence of diabetes among South Asians who have emigrated to other countries is associated with a persistence of the high-carbohydrate Indian diet [5,56]. As discussed above, these dietary changes may have interacted with underlying genetic susceptibility, developmental programming, and broader social and environmental changes to influence diabetes risk.

## 2. Discussion

### 2.1. A Dietary Link Between Type 2 Diabetes and Protein Malnutrition

The rise in type 2 diabetes associated with the ingestion of a carbohydrate-rich diet high in sugar and rice is similar to the rise in diabetes observed in Nauru with the introduction of sugar in the 1920s, as reported by the historian and anthropologist Jared Diamond [6]. However, a contrasting aspect is that severe obesity also developed in Nauru, while the appearance of diabetes in India, while commonly associated with mild to moderate obesity, could also be associated with malnutrition and low body weight (sometimes referred to as “tropical diabetes” [27,57,58,59].

One reason may be the high frequency of famines that have occurred in India over the centuries, often triggered by failure of the monsoons with drought. British reports from the 1800s documented the major impact on nutrition and survival [60,61]. Another explanation could be that the Hindu diet was normally low in protein. In its most severe form, protein malnutrition coupled with high sugar/carbohydrate diet results in the condition kwashiorkor. Kwashiorkor was not uncommon among children in India in the early twentieth century. A classic example resulted from the substitution of cow’s milk with rice water or rice milk, providing energy exclusively from carbohydrate without the amino acids necessary for growth and metabolic homeostasis [62].

**Figure 2:**
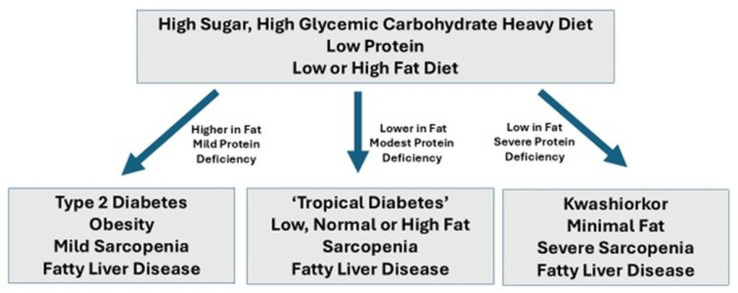
Hypothesized spectrum linking protein deficiency and diabetes phenotypes. This conceptual model illustrates a proposed relationship among kwashiorkor, type 5 diabetes, and obesity-associated type 2 diabetes through varying combinations of carbohydrate exposure, protein insufficiency, adiposity, and metabolic dysfunction. The model is intended as a heuristic framework and does not imply established causality. Additional mechanistic and longitudinal studies are needed to validate or refute this hypothesis.

We propose, as a working hypothesis, that kwashiorkor, type 5 diabetes, and obesity-associated type 2 diabetes may represent points along a spectrum of metabolic vulnerability influenced by varying degrees of carbohydrate excess, protein insufficiency, and developmental undernutrition (Figure 2). This conceptual framework is intended to generate hypotheses for future investigation rather than to imply a proven causal continuum. Kwashiorkor is classically associated with alopecia, dermatitis, muscle wasting, and severe pediatric protein deficiency, whereas type 2 diabetes is generally characterized by insulin resistance, adiposity, and later-life onset. Type 5 diabetes may occupy an intermediate position, reflecting chronic undernutrition, impaired β-cell function, and metabolic dysfunction without marked obesity. While shared metabolic features such as fatty liver, impaired glucose tolerance, high circulating fatty acids, and altered insulin secretion have been described across these conditions, the biological relationships among them remain incompletely understood and require further mechanistic study [63,64,65,66,67,68].

### 2.2. Carbohydrate Excess and Protein Malnutrition as Central Dual Contributors

Accordingly, growing evidence supports the concept that carbohydrate excess and protein malnutrition act as dual, interacting nutritional drivers of metabolic dysfunction in South Asian populations. Contemporary dietary assessments from the ICMR–INDIAB survey demonstrate that a substantial proportion of total energy intake comes from refined carbohydrates, particularly polished white rice and added sugars, while protein intake often remains below recommended levels for metabolic health [29,54,69]. Such carbohydrate-heavy diets promote postprandial hyperglycemia and hyperinsulinemia, stimulate hepatic de novo lipogenesis, and increase endogenous fructose production, thereby contributing to hepatic steatosis, insulin resistance, and related metabolic abnormalities [16,70,71].

When superimposed on chronically inadequate protein intake, these effects are amplified. Protein insufficiency reduces lean mass accretion, alters hepatic lipid export, and increases susceptibility to fatty liver and metabolic dysfunction [16,72]. Reviews of NAFLD and MASLD consistently note that low dietary protein, particularly in high-carbohydrate diets, contributes to greater hepatic triglyceride accumulation and impaired metabolic flexibility, whereas adequate protein intake tends to be protective [73,74,75,76,77]. Collectively, these findings indicate that excess carbohydrate exposure combined with insufficient protein intake serves as an important nutritional driver of metabolic disease in South Asian populations.

### 2.3. Linking Kwashiorkor, the “Thin-Fat” Phenotype, and Visceral Adiposity

Kwashiorkor, traditionally viewed as a severe form of protein–energy malnutrition, displays metabolic abnormalities such as fatty liver, endocrine dysfunction, impaired glucose tolerance and relative insulin deficiency that overlap with features of type 2 diabetes despite the absence of obesity [78,79,80,81,82]. This phenotype mirrors a broader “thin-fat” pattern well described in South Asians across the life course, beginning with the thin-fat Indian neonate and persisting into adulthood as disproportionate visceral and hepatic fat despite a relatively low BMI [30,31,32,83,84].

Studies from rural India show that infants are often born small but with relative adiposity, with preserved subcutaneous fat and lower lean mass—the “thin-fat Indian baby” that is strongly associated with later insulin resistance [81]. As adults, South Asians exhibit increased visceral and hepatic fat for any given BMI, develop type 2 diabetes at younger ages and lower BMI, and demonstrate earlier metabolic inflexibility than White Europeans [30,31,32,84]. This combination of reduced muscle mass and preferential ectopic fat storage closely parallels, though in milder form, the metabolic disturbances observed in kwashiorkor.

Together, these patterns support a continuum of metabolic vulnerability extending from severe malnutrition to modern type 2 diabetes. Within this continuum, inadequate protein intake and high carbohydrate exposure may shift individuals toward a sarcopenic, visceral-fat-dominant phenotype with high metabolic risk even in the absence of obesity [30,85], a pattern that closely resembles type 5 diabetes, a form of diabetes associated with chronic undernutrition, impaired β-cell function, and the relative preservation of insulin sensitivity.

### 2.4. Implications for Type 5 Diabetes and Global Recognition

Early clinical descriptions of diabetes in tropical regions documented lean individuals with atypical metabolic features [53,54,55,56], and subsequent physiological studies demonstrated impaired insulin secretion in the setting of protein–calorie deficiency [60], suggesting a biologically distinct entity. Building on these observations, the WHO formally classified this condition as malnutrition-related diabetes in 1985 [86]. However, this category was later removed from WHO classification in 1999 due to insufficient evidence establishing undernutrition as a causal factor [87].

More recent studies from low- and middle-income populations have renewed interest in this phenotype, describing individuals with low BMI, reduced insulin secretion, and relatively preserved insulin sensitivity, distinguishing them from classical type 1 and type 2 diabetes [88,89,90]. Building on this evolving evidence base, a 2025 international consensus statement (Vellore Declaration) has proposed the term type 5 diabetes to describe this condition and emphasized its defining features, including absence of autoimmunity, predominant β-cell dysfunction, and strong associations with early-life and chronic undernutrition [91].

Within this framework, kwashiorkor may represent the severe end of protein deficiency-related metabolic dysfunction, whereas the thin-fat phenotype observed across South Asian populations may represent a more chronic and subclinical manifestation. In contrast to classical obesity-associated type 2 diabetes, this form of diabetes is hypothesized to arise from long-standing macronutrient imbalance and developmental undernutrition, leading to reduced β-cell reserve and disproportionate metabolic risk at low BMI.

### 2.5. Protein-Sparing, Carbohydrate Excess, and Metabolic Risk

The protein-sparing hypothesis provides a mechanistic link between high-carbohydrate, low-protein diets and the metabolic abnormalities observed across South Asian populations. When dietary protein is limited, ingested carbohydrates are preferentially oxidized while lean tissue synthesis is constrained. Repeated high-glycemic carbohydrate loads stimulate hyperinsulinemia and hepatic de novo lipogenesis, increasing endogenous fructose production through the polyol pathway [71]. Experimental studies demonstrate that endogenous fructose can directly induce steatosis, mitochondrial stress, and insulin resistance even without excessive caloric intake [72].

At the same time, inadequate protein intake compromises hepatic VLDL export, reduces skeletal muscle mass, affects the primary site of insulin-mediated glucose disposal and may impair β-cell resilience, amplifying the metabolic consequences of carbohydrate excess [74,85,92,93,94]. Animal and human studies show that low-protein, high-carbohydrate diets exacerbate hepatic triglyceride accumulation and insulin resistance, whereas higher-protein diets improve metabolic outcomes [92,93,94].

These mechanistic observations align with population-level dietary patterns in India, where refined cereals and sugars predominate and protein intake remains marginal [69,70,95]. Under these conditions, the protein-sparing action of carbohydrate becomes maladaptive, favoring hepatic fat accumulation and progressive metabolic deterioration.

### 2.6. Gut Microbiota, Functional Foods, and Lifestyle-Based Approaches to Diabetes Prevention

Emerging evidence suggests that the relationship between dietary patterns and diabetes extends beyond macronutrient composition to include interactions with the gut microbiota. Diets rich in dietary fiber, legumes, fruits, vegetables, and other minimally processed plant foods promote microbial diversity and the production of short-chain fatty acids (SCFAs), including butyrate, acetate, and propionate, which have been associated with improved insulin sensitivity, reduced inflammation, enhanced gut barrier function, and better metabolic health [81,96,97,98]. Conversely, dietary patterns characterized by high intakes of refined carbohydrates, added sugars, and ultra-processed foods may promote gut dysbiosis, increased intestinal permeability, endotoxemia, and chronic low-grade inflammation, all of which have been implicated in the pathogenesis of type 2 diabetes and other non-communicable diseases (NCDs) [81,95,96,97]. Importantly, dietary composition appears to be a major determinant of microbiome structure and function. Thus, the high-carbohydrate, relatively low-protein dietary patterns historically observed in parts of South Asia may influence microbial ecology in ways that contribute to metabolic dysfunction, although causal relationships remain incompletely understood. Functional foods and nutraceuticals, including fiber-rich foods, fermented foods, polyphenol-rich plant products, and bioactive compounds such as curcumin and berberine, have shown potential metabolic benefits through effects on glucose metabolism, inflammation, and insulin sensitivity. When combined with regular physical activity, which enhances insulin sensitivity and preserves skeletal muscle mass, these dietary strategies may represent valuable adjunctive approaches for improving metabolic health and reducing the burden of diabetes and related NCDs. While further research is needed, the modulation of the gut microbiota through diet offers a promising mechanistic link between nutrition and metabolic disease prevention.

### 2.7. Alternative Contributors to the Diabetes Epidemic in India

Although this narrative review emphasizes the potential contribution of refined carbohydrates, sugar, and protein insufficiency, the diabetes epidemic in India is undoubtedly multifactorial. Urbanization, reduced physical activity, increased sedentary behavior, sleep disruption, environmental pollution, socioeconomic transitions, and the globalization of dietary patterns have all been associated with increasing diabetes prevalence [28,49,50,65]. Likewise, rising caloric intake, increased consumption of ultra-processed foods, and changes in dietary fat composition may contribute to obesity, insulin resistance, and metabolic dysfunction. Fructose may exert important metabolic effects through pathways involving hepatic lipogenesis and endogenous fructose production; however, these effects likely interact with total energy intake, adiposity, dietary fat intake, and microbiome alterations. Therefore, the historical associations described in this review should not be interpreted as evidence that sugar or fructose alone explains the diabetes epidemic in India, but rather as one potentially important component within a broader network of interacting biological, environmental, and societal factors.

## 3. Conclusions

India is the birthplace of sugar and was one of the first places where obesity and diabetes emerged. Nevertheless, diabetes remained at a relatively low prevalence until the British initiated the production of refined sugar and white, polished rice. The coexistence of high sugar and starch consumption with relatively low protein intake may represent one of several factors contributing to the diabetes epidemic in India. This historical and nutritional context helps explain the coexistence of obesity, diabetes, and malnutrition within the same populations, families, and even individuals. It also provides a unifying framework linking type 2 diabetes, insulin resistance, fatty liver, sarcopenia, and features of chronic undernutrition across the life course.

Collectively, these observations suggest that metabolic disease in South Asia is not solely a consequence of excess energy intake, but of macronutrient imbalance, with carbohydrate excess and protein scarcity functioning as interacting metabolic stressors. In this context, a subset of diabetes in South Asian populations may represent a modern manifestation of type 5 diabetes, reflecting the long-term metabolic consequences of chronic nutritional imbalance and undernutrition across the life course.

This emerging framework has important implications for diabetes prevention and treatment in India and other South Asian regions. First, interventions focused exclusively on reducing total calories or weight loss may overlook the central role of macronutrient composition. Strategies that lower refined carbohydrate intake while raising high-quality protein consumption may be more effective in reducing insulin resistance, mitigating hepatic fat accumulation, and improving overall metabolic health. Second, recognition of the thin-fat phenotype underscores the need for early screening and metabolic risk assessment in individuals who may appear normal weight by conventional criteria. Finally, given the persistent dominance of cereal-based diets in India, national nutrition programs may need to shift the focus toward increasing affordable protein sources to counteract this longstanding imbalance. These insights argue for a broader re-evaluation of nutritional recommendations in South Asia, emphasizing carbohydrate reduction and protein adequacy as foundational components in addressing the region’s diabetes epidemic.

## 4. Limitations

Several limitations should be considered when interpreting this narrative review. First, much of the evidence presented is historical, ecological, observational, or circumstantial in nature and therefore cannot establish causality. Second, dietary exposures, nutritional status, and diabetes diagnoses were often incompletely characterized in historical reports. Third, the proposed relationships among carbohydrate excess, protein insufficiency, kwashiorkor, type 5 diabetes, and type 2 diabetes remain hypothetical and require prospective mechanistic validation. Fourth, the historical rise in diabetes likely reflects multiple interacting influences, including genetics, urbanization, socioeconomic development, reduced physical activity, environmental exposures, and broader dietary transitions. Accordingly, the hypotheses presented here should be viewed as a framework for future investigation rather than definitive explanations for the diabetes epidemic in South Asia.

## Data Availability

No new data were created or analyzed in this study. Data sharing is not applicable to this article.
